# Effect on consumers’ sustainable purchase intention of dietary supplement purine labeling

**DOI:** 10.3389/fnut.2025.1526713

**Published:** 2025-06-04

**Authors:** Dajun Yang, Gui Gui, Yisong Yao, Xiong Ke

**Affiliations:** ^1^Key Laboratory of Digital-Intelligent Disease Surveillance and Health Governance, North Sichuan Medical College, Nanchong, China; ^2^Sichuan Provincial Primary Health Service Development Research Center, North Sichuan Medical College, Nanchong, China; ^3^School of Management, North Sichuan Medical College, Nanchong, China; ^4^School of Clinical Medicine, North Sichuan Medical College, Nanchong, China

**Keywords:** dietary supplements, purine labeling, sustainable purchase intention, health consciousness, fear of risk, disease threat, labeling theory

## Abstract

Previous studies have shown that nutritional labeling of dietary supplements plays a pivotal role in shaping consumer behavior. Nevertheless, few studies have focused on the relationship between purine labeling and consumers’ sustainable consumption intentions. To address this research gap, this study recruited 1,786 participants through six experiments to explore the effects of purine labeling on consumers’ sustainable purchase intentions, underlying mechanisms, and boundary conditions. The results showed that purine labeling significantly enhanced consumers’ intention to continue purchasing dietary supplements (Experiment 1); furthermore, health consciousness mediated the relationship between purine labeling and sustainable purchase intention (Experiment 2); further, the interaction between the threat of disease and purine label had significant effects on consumer sustainable purchasing (Experiment 3); finally, the moderating effect of fear of risk on the relationship between purine labeling and consumer sustainable purchase intention (Experiment 4). Immediately following this, this study demonstrated the mediating role of perceived gout susceptibility (Experiment 5) and the moderating role of the connection of nature (Experiment 6). The strengths of this study include its comprehensive approach, which consists of six experiments that explore the effects of purine labeling systematically. The large sample size and the use of mediation and moderation analyses provided robust evidence for the proposed relationships. However, this study also has some limitations, such as the generalizability of the findings being limited by the manipulated environment, the self-reported sustainable purchasing intention are biased, and the cross-sectional study not demonstrating how purchase intention changes over time. This study advances our understanding of the relationship between dietary supplement purine labeling and consumer purchase decisions and provides empirical support for marketing strategies, especially in health consciousness and environmental sustainability. In addition, this study provides new insights into how purine labeling further influences consumers’ purchase intentions through factors such as health consciousness disease threat and fear of risk, contributing to developing more effective labeling design and promotional strategies to promote sustainable consumer behavior.

## 1 Introduction

The global population is shifting toward healthier lifestyles, driven by both policy interventions and mass health perceptions (1–3). Therefore, governments are increasingly advocating for healthier diets in response to the increase in non-communicable diseases (NCDs) such as cardiovascular disease, diabetes and gout (4). One important initiative is the introduction of labeling systems to provide detailed nutritional information, including purine content, on dietary supplements. Purines are a metabolic product (5), with approximately 80% of endogenous purines coming from the oxidative breakdown of nucleic acids and 20% being exogenous purines from food intake (6). In the human body, purines are metabolized into uric acid (7). Hyperuricemia, characterized by elevated blood uric acid levels, has significant health implications (8). It is the primary clinical feature of gouty arthritis and is also associated with the development of uric acid kidney stones and cardiovascular diseases (9). However, numerous studies have indicated that certain dietary supplements, such as those containing animal-derived nucleic acids or yeast extract, are notably high in purines (10). For instance, supplements like some energy drinks, certain protein powders, and specific vitamin B-complex supplements have been identified to have elevated purine content (11–14). It is important to specify these examples to clarify that not all dietary supplements possess this characteristic. In today’s highly competitive and fast-paced society, people’s physical state is mainly in a sub-healthy state (15). Many people are becoming aware that dietary supplements can be used as a supplement for nutritional deficiencies (16). Although previous studies have extensively examined the labeling of dietary supplements (e.g., calorie, sugar content labeling, fat labeling) (17, 18), previous studies have largely ignored the impact of purine labeling of dietary supplements on consumer behaviors, in particular on sustainable purchase intentions. Therefore, understanding the purine labeling of dietary supplements and its impact on consumers’ sustainable purchase intentions is crucial for developing effective marketing strategies and catering to specific consumer needs.

Sustainable purchase intention is the tendency and willingness of consumers to consider the long-term environmental, social and economic impacts of their consumption behavior (19, 20). To be sure, a large body of literature on consumer sustainable purchase intention has driven consumer sustainable purchase intention to become a research hotspot and trend in the marketing field (21, 22). However, previous studies have focused on the relationship between food labeling and consumer purchase intention (23, 24). For example, Khan et al. (25) found that sugar-free labeled food products are more likely to stimulate consumers’ purchase intention. However, research on how labeling of nutritional products stimulates consumers’ purchase intentions is still essentially flawed. A recent study focused on high-pressure work groups showing a strong preference for dietary supplements with psychological ownership labels (26). However, the relationship between the purine labeling of dietary supplements and consumers’ sustainable purchase intentions has not received sufficient attention, and its theoretical value has not been fully recognized. This is because the transparency of purine content in purine labeling can help consumers make scientific purchasing decisions based on their health conditions. More importantly, for patients with gout and hyperuricemia, informative and transparent purine labeling is a key factor in managing their health status and preventing disease episodes. However, concerning the current literature survey, the mechanisms and boundary conditions by which the purine labeling of dietary supplements influences consumers’ sustainable purchase intentions are unclear. Therefore, does the purine labeling of dietary supplements positively affect on consumers’ sustainable purchase intentions? What are the internal mechanisms by which purine labeling of dietary supplements influences consumers’ sustainable purchase intentions?

In order to fill the research gap on the purine label of dietary nutritional supplements on consumers’ sustainable purchase intention, this study constructed a theoretical framework to explore the impact of the label of dietary nutritional supplements on consumers’ sustainable purchase intention and its internal mechanism and boundary conditions. In line with labeling theory, we focus on how information transmission in dietary supplements, specifically purine content information, guides consumer choice (27). Previous studies have discussed the impact of various nutrition labels on purchasing behavior (28), such as sugar-free (29), calorie labels (30), fat labels (31) and so on. However, purine labels have been neglected. Firstly, this study proposes the impact of purine labels on consumers’ sustainable purchase intention. Our study contributes to the existing literature by combining purine labeling of dietary supplements with sustainable purchase intentions and exploring the mechanisms linking the two. Immediately following, we examined the mediating effect of health awareness, which further explains the effectiveness of dietary supplement purine labeling in increasing consumer sustainable purchase intentions. We then analyze the disease threat and risk fear interactions that can better clarify the boundary conditions between the purine label of dietary supplements and the sustainable purchase intention of consumers. Therefore, the theoretical contribution of this study focuses on providing a systematic explanation and understanding of how purine labeling of dietary supplements combines with consumers’ health awareness, disease threat and risk fear to influence consumers’ sustainable purchasing intentions. The study also provides valuable guidelines for merchants to improve consumers’ sustainable purchase intentions.

## 2 Literature review and theoretical derivation

### 2.1 Application of labeling theory in marketing

Labeling theory was initially developed by sociologist Howard Becker in the 1960s to explain how social labels influence individual behavior (32–34). The core idea of labeling theory is that when society assigns particular labels to certain individuals or groups, these labels may shape an individual’s self-perception, which in turn influences the way he or she behaves (32, 35). In marketing, labeling theory is widely used to explain how the social perceptions of a brand, product or individual can influence consumer attitudes and behaviors through product labels (36, 37). For example, Pagès et al. (38) found that when a product is labeled as “alcohol-free,” consumers tend to perceive the product as more health-conscious, thus increasing their purchase intentions.

A reading of the literature reveals that there has been a significant amount of research exploring the application of labeling theory in marketing (39). Many scholars have focused on the ability of product labels to influence consumer perceptions of products significantly (40, 41). For example, Delmas and Lessem (42) found that the organic label made consumers willing to pay a premium for the product because of its strong association with high quality and health. In addition, Kühne et al. (43) explored the impact of ‘new product’ labels on consumers’ acceptance of innovation. They found that products with innovative labels were more likely to be perceived as cutting-edge and breakthrough technology products. Despite the wide application of labeling theory in marketing, some important research gaps remain. First, most current research focuses on the direct effects of labels on individual perceptions and behaviors (44). At the same time, there is a relatively limited exploration of how labels have indirect effects through complex social interactions and cultural contexts. Second, the differential manifestations of the label effect in different consumption contexts, cultural backgrounds and communities have not been thoroughly investigated, limiting the general applicability of the theory. Finally, most of the existing studies have focused on the simple association between specific types of labels and consumer behavior while neglecting the deeper psychological mechanisms and contextual factors moderating the label effect.

To address shortcomings of the above research, we propose the following solutions. First, by introducing health awareness as a mediating variable, we delve into how purine labeling affects consumers’ purchase decisions by influencing their health perceptions. This helps to provide a more in-depth understanding of the internal mechanism of the labeling effect and reveals the psychological process of consumers in making purchase decisions, thus providing a more comprehensive theoretical explanation. Second, by introducing disease threat and fear of risk as moderating variables, we explore how these situational factors affect the strength and direction of the labeling effect. This move can fill the lack of exploration of the differential manifestation of the labeling effect in existing studies, making the application of labeling theory in marketing practice more relevant and flexible. Finally, this study takes sustainable purchase intention as an outcome variable, which not only expands the research scope of the labeling effect, but also provides theoretical support for promoting sustainable consumption. This shift in perspective makes up for the lack of exploration of the long-term impact of the labeling effect in existing studies, and helps companies to better consider sustainable development goals in label design.

### 2.2 Purine labeling and other health labelings

As food labeling becomes increasingly important in modern food production and consumption, various health labels have been developed to guide consumer choices and meet specific dietary needs (45, 46). Purine labeling have recently gained attention due to their relevance to specific health conditions. Previous studies have focused on sugar labeling, organic labeling, calorie labeling, and allergen warning labeling, among others (47–50), but have not specifically differentiated these from purine labeling. This section compares purine labeling with other standard health labeling, highlighting their unique characteristics, advantages, and limitations to provide insights into their practical applications and potential improvements.

Purine labeling and sugar labels both address specific dietary needs: Purine labels are targeted at consumers with gout or kidney disease, while sugar labels aim to help consumers manage sugar intake, particularly those at risk of diabetes or obesity (18). However, their target audiences differ significantly. Purine labeling are more specialized, focusing on managing purine intake, which is crucial for preventing the accumulation of uric acid in the body. In contrast, sugar labeling have a broader applicability, as they provide information relevant to a vast population, including those who prefer low-sugar products (29). Regarding information specificity, purine labeling offer detailed information about purine content, enabling precise dietary control for their target audience. While sugar labeling are important, they typically provide less granular information, often indicating whether a product is low in sugar or contains added sugars. Compared to the broad applicability of sugar labeling, this specificity highlights purine labeling’ niche but precise nature.

The comparison between purine and organic labeling reveals that both cater to health-conscious consumers (51), though their focuses and implications differ. Organic labeling emphasize production processes and ingredient sourcing, ensuring that products contain no synthetic chemicals, artificial additives, or genetically modified organisms during growth or production (52). These labelings appeal to consumers who prioritize environmental sustainability, animal welfare, and perceived health benefits associated with organic products. On the other hand, purine labeling is primarily health-oriented, providing direct nutritional information to manage specific health conditions. While organic labelings encompass broader ethical and environmental considerations, purine labeling is confined to specific nutritional issues. This distinction underscores the complementary nature of these labelings: organic labelings address production practices and consumer values, whereas purine labeling address direct nutritional needs.

Calorie labeling is designed to assist with weight management and energy intake control (53, 54), serving a broad audience ranging from individuals aiming to lose weight to maintaining balanced diets. These labelings frequently appear on restaurant menus and packaged foods, offering consumers quick references to make informed decisions about their energy intake. In contrast, purine labeling serves a more specialized audience, focusing on dietary management for purine-related health issues. Calorie labels provide direct measurements of energy content (55), while purine labelings offer nuanced measurements of specific nutrients critical to certain health conditions. This comparison highlights the importance of tailoring food labels to meet the needs of different consumer groups—calorie labelings address universal concerns, while purine labelings fulfill specific medical needs.

Finally, comparing purine labelings and allergen warning labelings reveals both similarities and differences. Both labels are critical for consumer health and safety, providing essential information to prevent adverse health reactions (56, 57). In many countries, allergen warning labelings are legally mandated to protect individuals with life-threatening food allergies, such as those allergic to peanuts, tree nuts, milk, eggs, fish, shellfish, wheat, and soy (58). These labels ensure that allergic consumers can make safe food choices. Similarly, purine labelings serve a comparable purpose by providing critical information for individuals managing gout or kidney disease. However, unlike legally mandated allergen warning labelings, purine labelings are typically voluntary or advocated by health organizations. This distinction suggests that purine labelings could benefit from broader adoption and regulation to ensure their availability and consistency across food products. Specific comparisons are detailed in Table 1.

**Table 1:** Summary table of purine labeling and other labelings.

Comparison aspect	Primary target audience	Specific focus	Information specificity	Key benefit	Application scope
Purine labeling	Gout sufferers, kidney disease patients	Purine content in food	Detailed purine content, often numerical	Prevents uric acid buildup, manages gout	Specific to high-purine foods
Sugar labeling	General population, particularly those managing diabetes or obesity	Sugar content, particularly added sugars	General or categorical (e.g., “low in sugar”)	Helps control sugar intake, reduces diabetes risk	Applicable to most packaged and processed foods
Organic labeling	Health-conscious, environmentally aware consumers	Production practices, no synthetic additives or GMOs	Broad, encompassing production practices	Promotes sustainable, ethical production; perceived health benefits	Applicable to all food products meeting organic standards
Calorie labeling	General population, especially those managing weight	Energy content, caloric intake	Numerical calorie count	Assists with weight management	Applicable to all foods with calorie information
Allergen warning labeling	Individuals with food allergies	Presence of allergens	Presence or absence of allergens	Protects against life-threatening reactions	Applicable to all foods containing allergens

### 2.3 The impact of purine labeling on consumers’ sustainable purchase intention

As living standards improve, consumers are increasingly concerned about the composition of food and health products (59, 60). However, in the face of increasingly complex nutritional label information, consumers are often in a dilemma when choosing products. On the one hand, they want to obtain more health information through health labeling (61). Conversely, information overload and label comprehension difficulties may lead to decision-making fatigue, affecting consumers’ purchase intention (62). Among the many nutritional labeling, the purine labeling is often ignored by manufacturers of dietary supplements. However, this dilemma is particularly salient for consumers when purchasing dietary supplements with purine content. Consumers are conflicted about dietary supplements, whether they should prioritize looking for or considering purine-labeled dietary supplements to assess the potential health effects of the food, or whether they should prioritize focusing on analyzing the dietary supplements for the presence of purines. In reality, consumer perceptions of purine labeling are very limited. According to the Health Belief Model (HBM), we found that the stronger consumers’ perception of purine-related health risks, the higher their motivation to avoid high-purine products (63). However, among the existing studies, we found that few studies have proposed the concept of purine labeling and conducted relevant research. Therefore, we believe that purine labeling aims to help consumers understand the purine content of a product so that they can make more informed choices based on their health conditions. Such labeling provides important health information and can influence consumers’ purchasing decisions by guiding them to choose products that are better suited to their health needs.

Sustainable purchase intention reflects consumers’ recognition of product quality and brand trust, as well as their importance on environmental protection and healthy lifestyles (64, 65). Previous studies have shown that factors influencing sustainable purchase intention include brand image, product quality, perceived health risks, and consumers’ environmental awareness (66, 67). For example, in a study by Siraj et al. (68), sustainable labeling was found to contribute to consumers’ sustainable purchase intentions positively. Although this study focused on the relationship between labeling and consumers’ sustainable purchase intention, no logical relationship between purine labeling and consumers’ sustainable purchase intention has been found.

Purine labeling, as a health risk cue, can influence consumers’ health perceptions and thus their purchasing decisions. Between purine and sustainable purchase intention, a label enables consumers to make a clearer judgment on whether a particular product meets their health needs and motivates them to consistently choose these health-compliant products. This mechanism of action explains why purine labeling is effective in enhancing consumers’ sustainable purchase intentions, especially among those consumer groups that place a high value on health.

Based on the above analysis, we propose:

*H1*: Purine labeling of dietary supplements has a significant effect on consumers’ sustainable purchase intentions.

### 2.4 The mediating role of health consciousness

Health consciousness refers to an individual’s perceptions of his or her health status and behaviors and willingness to take action to maintain or improve health (69, 70). Research has shown that health consciousness is strongly associated with various health-related behaviors, including dietary choices, exercise habits and perceived health risks (71, 72). For example, Loebnitz and Grunert (73) noted that health consciousness not only influences an individual’s healthcare choices but also significantly affects consumers’ food choices and consumption behaviors. Specifically, consumers with higher health consciousness tend to be more inclined to choose products that improve or maintain their health, especially regarding diet (74). These consumers are more likely to choose foods low in fat, sugar, salt, fiber and natural ingredients (75). At the same time, increased health consciousness has prompted consumers to pay more attention to the ingredients in food products, especially those that may have a negative impact on health (76), such as trans fatty acids.

Choi et al. (77) research further suggests that health-conscious consumers are more likely to understand and interpret food labeling information, especially the health information involved in the labels. Such consumers are more likely to use the nutritional information on the labels as an important basis for purchase decisions (78). Specifically, consumers with high health consciousness also show a stronger tendency to avoid health risks and choose products that are more beneficial to the body (79). This phenomenon is particularly noticeable in the choice of dietary supplements, which, as products that supplement the body with essential nutrients, have become the core of consumers’ attention in terms of their ingredients and health benefits (80). Against this backdrop, health consciousness has not only increased consumer demand for dietary supplements, but also raised the importance they place on ingredient labeling (81).

Although the relationship between health consciousness and dietary supplement choice has been extensively discussed in existing studies (82), the specific role of purine labeling remains insufficiently explored. Based on the existing research, it is hypothesized that consumers with higher health consciousness may be more concerned about the purine content of dietary supplements. Health-conscious consumers are more sensitive to perceived health risks and prefer products that are lower in purines. Purine labeling plays an important messaging role in this process, helping consumers better assess a product’s health risks or benefits.

In this framework, health consciousness enhances consumers’ comprehension of purine labeling and influences their purchasing decisions. For example, consumers with high health consciousness may develop negative attitudes toward dietary supplements with high purine content while showing higher purchase intentions toward products with lower purine content (83). This health risk aversion behavior is also reflected in consumers’ sustainable purchase intentions (84). The study shows that consumers’ sustainable purchasing behavior is not only related to environmental consciousness but also closely related to health consciousness. Therefore, health consciousness ultimately affects consumers’ sustainable purchase intentions by influencing their interpretation of purine labeling.

Based on the above analyses, we propose the following hypotheses:

*H2*: Health consciousness mediates purine labeling in dietary supplements and consumers’ sustainable purchase intentions.

### 2.5 Disease threat interactions

Disease threat refers to consumers’ perception of a disease or health risk that motivates them to take preventive measures to reduce risk exposure (85, 86). The Extended Parallel Process Model (EPPM) proposed by Witte (87) states that when individuals perceive a health threat, they are more inclined to take protective actions to avoid the risk, especially when the severity and susceptibility of the threat are high. The model is widely used in consumer behavior research, to explain how individuals make decisions when faced with health risks (88). It has been shown that higher disease threat perception enhances consumers’ concern for healthy products and significantly affects their sensitivity to food labeling and ingredients (89). Recent research further suggests that disease threat plays a key role in driving consumers to choose healthier products, particularly in the nutraceutical and functional food markets (90).

In the context of purine labeling, risk aversion has become an important feature of consumer behavior in disease-threatening situations. Purines, a compound commonly associated with gout, pose a significant risk to consumers prone to the disease (91). Therefore, labeling the product’s purine content can enhance their awareness of the potential health risks associated with consumption (92). When consumers perceive a health threat, they show a stronger tendency to be risk-averse and are, therefore, more likely to choose low or no-purine alternatives. However, purine labeling may also serve as a risk management tool to help consumers who are highly concerned about their health to choose products that meet their health needs. Thus, purine labeling may have a dual impact on consumer purchasing decisions under the threat of disease, an impact that depends on the way consumers interpret health information and their perceived level of risk.

According to information processing theory, consumers usually adopt simplified cognitive strategies when faced with complex product information (93). When purine labeling is associated with disease threats, the message delivery effect will be more significant, especially among groups at high risk of diseases such as gout. Purine labeling not only helps them to avoid health risks but also enhances brand trust, thus increasing their willingness to make sustainable purchases (94). Consumers are more likely to remain motivated to make sustainable purchases when they perceive products as meeting their long-term health needs (95). Therefore, the standardization of purity labeling and information transparency plays an important role in enhancing consumers’ overall perception of nutritional products.

Based on the above analyses, we propose the following hypotheses:

*H3*: Disease threat significantly moderates purine labeling of dietary supplements and consumers’ sustainable purchase intentions.

### 2.6 The moderating role of fear of risk

Fear of risk is closely related to an individual’s perception of a potential threat or hazard (96, 97). This perception may arise from various factors, such as environmental, health, financial, or social risks, and significantly influences an individual’s decision-making process (98), especially in situations involving health and safety. In food consumption, fear of risk is closely linked to a high level of consumer concern about food safety (99). Research has shown that fear of risk substantially alters consumers’ purchasing behavior when faced with uncertainty about health threats (100).

The intensity and manifestation of fear of risk are moderated by several factors, including individual traits, cultural background, and risk cues in external messages (e.g., labeling or advertising) (100). Potential health risks have been the focus of attention in the selection of dietary supplements, especially for ingredients such as purines, which have been directly associated with diseases such as gout, which in turn increases consumer risk perceptions.

According to risk perception theory, individuals tend to develop a self-protective response when they become aware of potential risks, influencing their purchase decision-making process (101). Consumers’ awareness of health risks associated with ingredients such as purines in a dietary supplement purchase situation prompts them to pay more attention to product labeling (102). When product labels explicitly suggest the presence of purines, this direct risk cue triggers consumers’ fear of risk and influences their purchase behavior. Specifically, when consumers see purine risk cues, they tend to enhance their perception of health threats, reducing their purchases of purine-containing dietary supplements and shifting them toward alternatives with lower health risks.

Further, consumers, when faced with risky decisions, tend to favor the avoidance of potential losses rather than the pursuit of gains (103). When purine labeling suggests potential health risks, the fear of risk triggered can lead to enhanced avoidance behavior, i.e., avoidance of purine-containing products. Risk-averse consumers react more sensitively to such risk information and may choose alternative products or even avoid the purchase altogether. However, for those consumers with lower fear of risk, their behavior may be different, and they may continue to purchase purine-containing products or base their decisions on other factors such as price and brand.

Based on the above analysis, we propose the following hypotheses:

*H4*: Fear of risk has a significant moderating effect on the purine labeling of dietary supplements and consumers’ sustainable purchase intentions.

### 2.7 The mediating role of perceived gout susceptibility

Perceived gout susceptibility is an individual’s subjective judgment of their likelihood of developing gout based on their health status, family history, and health behaviors (104). Gout is a metabolic disorder closely associated with abnormal purine metabolism (105), characterized by elevated blood uric acid levels that trigger inflammatory responses in joints and soft tissues (106). Research has shown that the pathogenesis of gout is closely linked to dietary habits, lifestyle, and genetic factors (107). In recent years, perceived gout susceptibility has garnered significant attention from academia as an important concept in health psychology. Studies indicate that perceived gout susceptibility is crucial in health interventions (108). For instance, individuals with higher perceived gout susceptibility are more likely to engage in proactive health behaviors, such as adjusting their diet, reducing purine intake, and increasing physical activity (109). This highlights that perceived gout susceptibility is a key psychological factor in the prevention and management of gout and a critical driver influencing dietary choices and health behaviors (93). From a nutritional perspective, perceived gout susceptibility is also closely related to the use of dietary supplements. Individuals with higher perceived susceptibility are more inclined to choose supplements that help lower uric acid levels or improve metabolic conditions. This trend reflects how consumers actively manage their health status through diet and nutrition when facing the risk of chronic diseases. However, the accuracy of perceived gout susceptibility is closely tied to an individual’s knowledge level, health beliefs, and information-processing abilities.

Purine labeling information holds significant relevance for consumers with high perceived gout susceptibility. Studies have shown that consumers carefully examine the nutritional information on product labels when purchasing dietary supplements, especially components related to their health conditions (110). Individuals with higher perceived susceptibility are likelier to opt for products with lower purine content and exhibit greater sensitivity to purine labeling information (111). Consequently, at the cognitive level, these individuals are more attentive to purine labeling information and tend to interpret it more deeply. They are inclined to perceive products with lower purine content as safer and more aligned with their health needs. Simultaneously, at the behavioral level, individuals with high perceived susceptibility are more likely to purchase based on purine labeling information, avoiding products with higher purine content. Perceived gout susceptibility may also influence consumers’ perceptions and usage of purine labels through their health beliefs and behavioral intentions. Individuals with higher perceived susceptibility may be more inclined to trust the accuracy of purine labels and rely on them as a key factor in their purchase decisions. This trust further strengthens the relationship between perceived gout susceptibility and purine labeling.

Individuals with high perceived gout susceptibility typically exhibit a heightened health consciousness, which extends beyond personal health concerns to environmental and social awareness. For instance, they may prefer organic, non-hazardous, or biodegradable packaging for dietary supplements, as these products benefit personal health and positively impact the environment and society.

Perceived gout susceptibility indirectly influences purchase decisions by affecting how consumers perceive and use purine labeling. In this process, individuals with higher perceived susceptibility are more inclined to pay attention to purine labeling information and make purchase choices based on this information. This indicates that perceived gout susceptibility plays a critical mediating role in shaping consumer choices of dietary supplements. Furthermore, perceived susceptibility influences consumers’ health beliefs and behavioral intentions, affecting their willingness to make sustainable purchases. Individuals with high perceived susceptibility are not only more inclined to choose products with lower purine content but may also pay greater attention to the sustainability attributes of products. This dual focus further reinforces the impact of perceived susceptibility on sustainable purchasing intentions.

Based on the above analysis, we propose the following hypothesis:

*H5*: Perceived gout susceptibility mediates the relationship between purine labeling and sustainable purchasing intentions.

### 2.8 The moderating role of connection of nature

Connection of nature refers to an individual’s perception of a close and unified relationship with the natural environment (112). In 1984, Wilson proposed the “biophilia” hypothesis, suggesting that humans have an inherent and profound connection with nature, rooted in the co-evolutionary process between humans and their natural surroundings (113). Subsequent research further expanded the concept of connection of nature, defining it as a subjective experience that includes love for nature, dependence on it, and a sense of harmony with the natural world (114). Studies have demonstrated that connection of nature is not only closely associated with individual mental health but also significantly influences environmental behaviors and attitudes (115). For instance, high levels of connection of nature are often correlated with stronger environmental consciousness, more proactive eco-friendly behaviors, and a greater sense of ecological responsibility (116). Thus, the connection of nature, as a psychological and emotional state, is a critical bridge for human interaction with nature.

As consumer demand for healthy foods and natural products continues to grow, the role of connection of nature about purine labeling has become increasingly important. First, individuals with a high sense of connection of nature are more inclined to choose products with natural, eco-friendly, or sustainable attributes (117), making them more likely to be drawn to purine-labeled products. Second, individuals with a strong connection of natures tend to be more sensitive to the concept of “naturalness” and are more likely to trust natural ingredients’ safety and health benefits (118), thereby exhibiting stronger trust in purine labeling. However, the relationship between connection of nature and purine labeling may not be linear, as it is influenced by the complex cognitive and emotional perceptions individuals have of nature. For example, when the connection of nature is low, purine labeling may fail to trigger purchasing interest, while when the connection of nature is high, purine labeling may become a key decision-making factor. Therefore, the connection of nature plays a significant role in the effectiveness of purine labeling on dietary supplements.

The connection of nature as a psychological state has been widely studied and validated as a mechanism influencing sustainable purchasing intentions (119). According to theoretical and empirical research, individuals with a high sense of connection of nature typically exhibit stronger environmental attitudes, greater ecological responsibility, and a greater willingness to contribute to environmental protection (120). The intrinsic mechanisms underlying this relationship primarily include the connection of nature enhances ecological awareness (121), making individuals more attentive to the environmental impacts of products (122); connection of nature fosters intergenerational thinking, leading individuals to support sustainable development more readily; and connection of nature strengthens environmental responsibility (123), thereby increasing the likelihood of purchasing green products. Additionally, research has shown that individual values and consumption culture may moderate the relationship between the connection of nature and sustainable purchase intentions (124), but overall, the connection of nature is a significant positive predictor of sustainable purchasing intentions.

Connection of nature may also indirectly influence sustainable purchasing intentions by enhancing the perceived effects of purine labeling. Specifically, when consumers have a high sense of connection of nature, purine labeling may further stimulate their preference for and trust in natural products, strengthening their purchasing intentions. Conversely, when the connection of nature is low, the impact of purine labeling may be weaker or even negligible. According to Self-Concept Theory (125), individuals with a strong connection of nature are more likely to view being nature-friendly as part of their self-identity, making them more susceptible to the influence of nature-related information. Empirical studies have also shown that the interaction between the connection of nature and purine labeling has a significant impact on sustainable purchasing intentions, particularly in product categories such as health foods and dietary supplements (126).

Based on the above analysis, we propose the following hypothesis:

*H6*: The connection of nature moderates the relationship between purine labeling on dietary supplements and consumers’ sustainable purchasing intentions.

**Figure 1:**
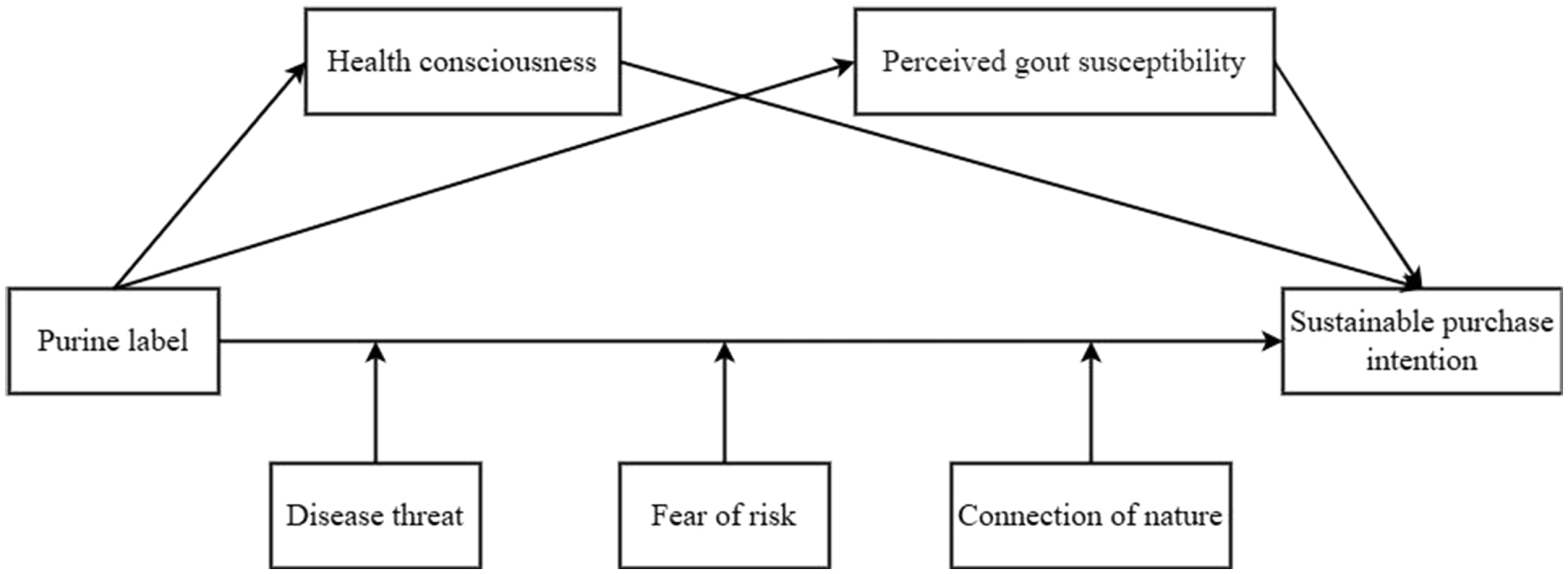
Theoretical model frame diagram.

The conceptual model is shown in Figure 1.

## 3 Overview of the study

**Figure 2:**
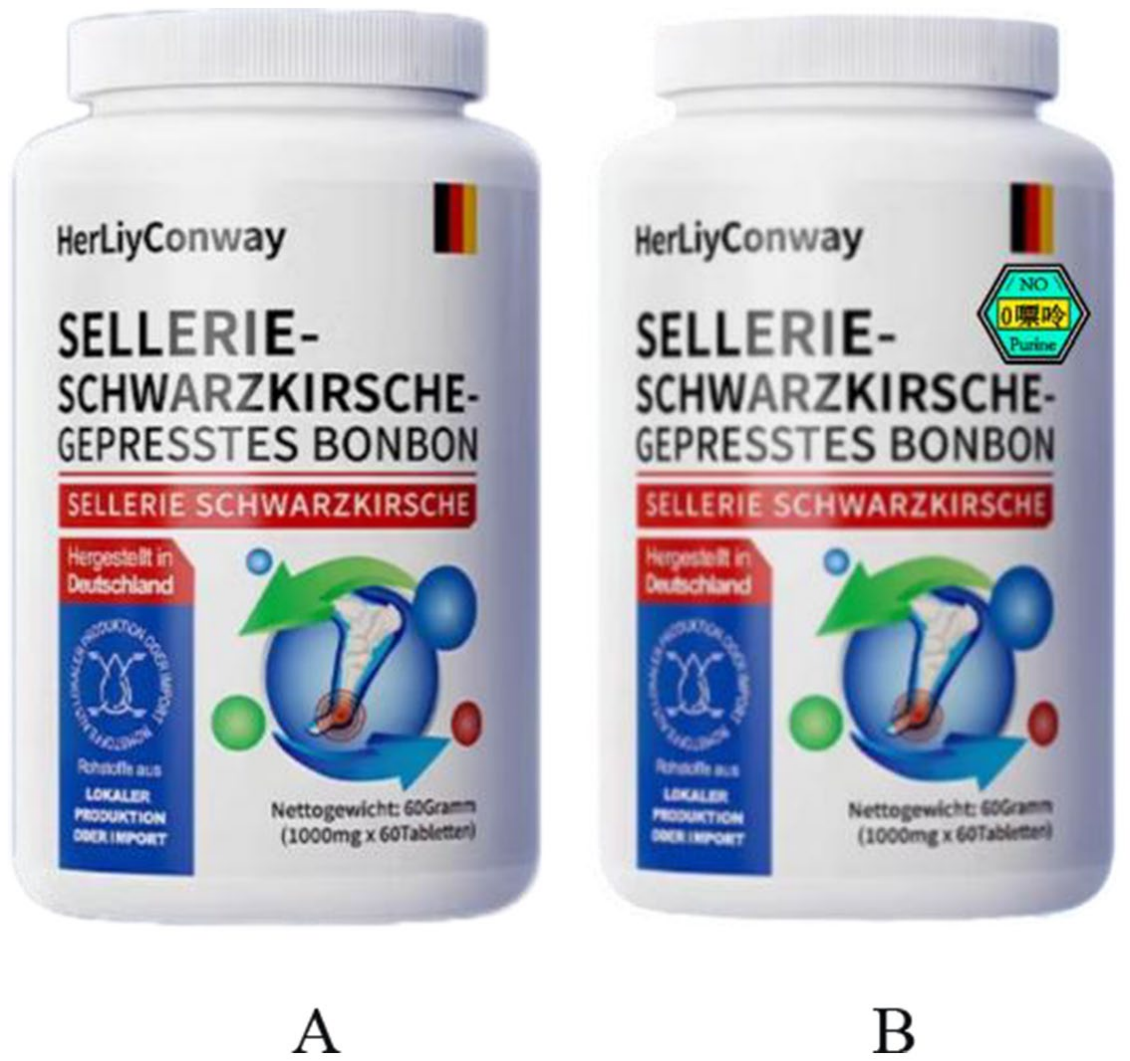
Experimental plot of purine labels of dietary supplement. **(A)** is the experimental stimulus picture of the control group (conventional group) and **(B)** is the experimental stimulus picture of the experimental group (purine group).

Six experiments were conducted to test the above six research hypotheses. In Experiment 1, we verified the effect of purine labeling of dietary supplements (purine group vs. conventional group) on consumers’ sustainable purchase intentions to test Hypothesis H1; in Experiment 2, we analyzed the mediating effect of health awareness on the purine labeling of dietary supplements (purine group vs. conventional group) and on consumers’ sustainable purchase intentions to test Hypothesis H2; and in Experiment 3, we explored the effect of the interaction of disease threat (high vs. low) and purine labeling of dietary supplements (purine group vs. conventional group) on consumers’ sustainable purchase intentions, testing Hypothesis H3; in Experiment 4, we illustrate the moderating effect of fear of risk (high vs. low) on purine labeling of dietary supplements (purine group vs. conventional group) and consumers’ sustainable purchase intentions, testing Hypothesis H4. In Experiment 5, we tested the mediating effect of perceived gout susceptibility on purine labeling of dietary supplements and consumers’ sustainable purchase intention, testing Hypothesis H5. In Experiment 6, we examined the moderating effects of connection of nature on purine labeling of dietary supplements and consumers’ sustainable purchase intention. To further implement the purine labeling manipulation, we plotted different experimental stimuli and wrote different stimulus materials to enhance the accuracy of the experiment. Meanwhile, we designed two different contextual materials to manipulate disease threats. The experimental plot of purine labels of dietary supplements,^1^ as shown in Figure 2. The ingredients of this product are celery, parsley, cherries, poria powder, mulberry leaf, turmeric, chicory and liquorice, which help to alleviate hyperuricemia and inhibit xanthine oxidase activity (127–133), and the origin of this product is Germany. This dietary nutritional supplement is a low-purine food, and the “No purine” label was set for experimental purposes only.

In Table 2, the “no purine” labeling, “low purine” labeling, “medium purine” labeling, and “high purine” labeling designed in this study are shown. We chose the “no purine” labeling because this study aimed to clearly distinguish whether the presence of purines in a product has a positive effect on the consumer’s intention to buy sustainable products. However, we chose other purine labeling to explore the impact of dietary supplements’ type of purine labeling on consumers’ sustainable purchase intention. Therefore, we performed experiments using only the “no purine” labeling.

**Table 2:** Four types of purine labeling and typical foods.

Purine label types	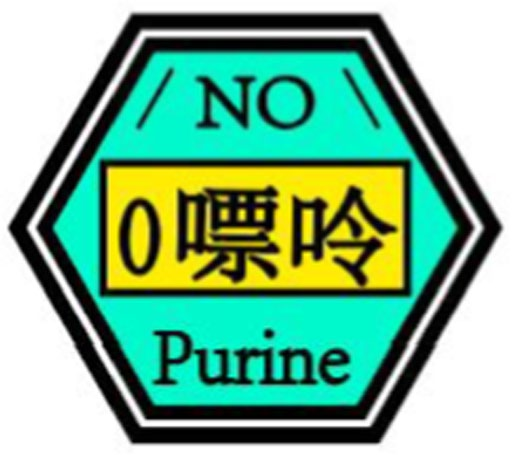	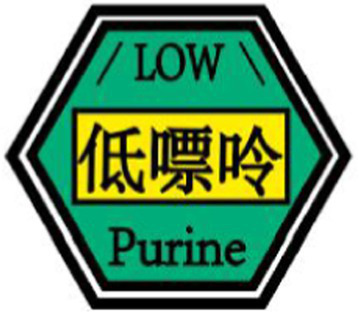	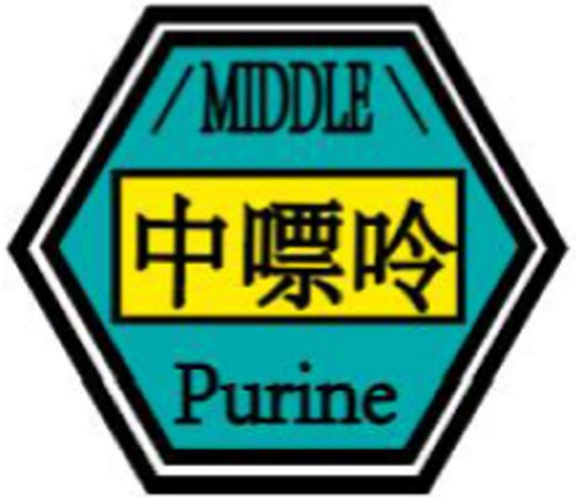	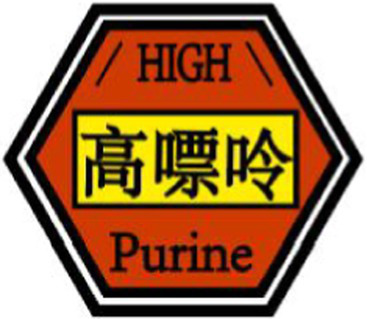
Product purine content (mg/100 g)	<30	<75	75 ~ 150	150~
Common foods	Dairy products, cheese, eggs, fruits, cocoa, carrots, tea, sea cucumbers, soy milk, corn, etc	Asparagus, cauliflower, green beans, green beans, kidney beans, mushrooms, spinach, peas, etc	Lentils, carp, sea bass, smoked ham, pork, beef, quails, rabbits, etc	Liver, brain, kidney, beef and mutton tripe, sardines, thick broth, etc

The experimental framework associated with these six studies is shown in Table 3. The demographic information of the six experiments is shown in Table 4. The scales used in the six experiments are shown in Table 5.

**Table 3:** Organization and description of statistical analysis of the experiments.

Experiment	Purpose	Independent variable	Dependent variable	Mediators	Moderator	Methods		Results
Experiment 1	To test for main effect (H 1)	Purine label	Sustainable purchase intention	-	-	ANOVA		Supported H1
Experiment 2	To test the mediation effect of health consciousness (H2)	Purine label	Sustainable purchase intention	Health consciousness	-	ANOVA	PROCESS 4	Supported H2
Experiment 3	To test the interaction effect of disease threat (H3)	Purine label	Sustainable purchase intention	-	Disease threat	ANOVA	TWO-WAY-ANOVA	Supported H3
Experiment 4	To test the moderating effect of fear of risk (H4)	Purine label	Sustainable purchase intention	-	Fear of risk	ANOVA	PROCESS 1	Supported H4
Experiment 5	To test the mediation effect of perceived gout susceptibility (H5)	Purine label	Sustainable purchase intention	Perceived gout susceptibility	-	ANOVA	PROCESS 4	Supported H5
Experiment 6	To test the moderating effect of connection of nature (H6)	Purine label	Sustainable purchase intention	-	Connection of nature	ANOVA	PROCESS 1	Supported H6

**Table 4:** Demographic information of the six experiments.

Variable		Gender		Age				Education background						
Item		Male	Female	18–25 years old	26–40 years old	41–60 years old	Over 61 years old	Primary school	Junior high school	Technical secondary school.	High school	Undergraduate college	Postgraduate	Doctor-postgraduate
Experiment 1 (*N* = 184)	Frequency	90	94	21	54	54	55	9	26	17	26	36	32	38
	Proportion	48.9%	51.1%	11.4%	29.3%	29.3%	29.9%	4.90%	14.1%	9.2%	14.1%	19.6%	17.4%	20.7%
Experiment 2 (*N* = 275)	Frequency	142	133	73	85	80	37	13	21	17	36	65	66	57
	Proportion	51.6%	48.4%	26.5%	30.9%	29.1%	13.5%	4.7%	7.6%	6.2%	13.1%	23.6%	24%	20.7%
Experiment 3 (*N* = 500)	Frequency	250	205	225	220	27	28	29	25	24	21	203	172	25
	Proportion	50%	50%	45%	44%	5.4%	5.6%	5.8%	5%	4.8%	4.2%	40.6%	34.4%	5.2%
Experiment 4 (*N* = 270)	Frequency	132	138	71	85	54	60	8	7	7	7	146	56	39
	Proportion	48.9%	51.1%	26.3%	31.5%	20%	22.2%	3%	2.6%	2.6%	2.6%	54.1%	20.7%	14.4%
Experiment 5 (*N* = 281)	Frequency	140	141	74	85	67	58	13	21	43	19	70	61	54
	Proportion	49.8%	50.2%	25.3%	30.2%	23.8%	20.6%	4.6%	7.5%	15.3%	6.8%	24.9%	21.7%	19.2%
Experiment 6 (*N* = 276)	Frequency	142	134	105	81	37	53	24	51	30	28	62	43	38
	Proportion	51.4%	48.6%	38%	29.3%	13.4%	19.2%	8.7%	18.5%	10.9%	10.1%	22.5%	15.6%	13.8%

**Table 5:** Scales used in the six experiments.

Variable	Measurement items	Scale source
Demographic information	What is your gender?	
	What’s your age?	
	What is your education background?	
Health consciousness	I care deeply about your health.	Gould (136)
	I regularly reflect on my health.	
	I are usually very mindful of how I feel inside about my health.	
Fear of risk	I become nervous or anxious when I see disease-related information on dietary supplements.	Rather (141)
	My heart races or palpitates when I think of diseases caused by purines.	
	I cannot sleep because I’m worried about the purine content of my dietary supplements.	
Emotion	I current emotional state is positive (positive, happy, cheerful).	Kuang et al. (140)
Consumption habit	Do you choose to buy regular dietary supplements out of habit?	Saba and Di Natale (137)
Perceived gout susceptibility	I’m unlikely to get gout, hyperuricemia, or other kidney disease, even though it’s highly susceptible.(reverse-scored)	Duncan et al. (142)
	My immune system protects me from most illnesses that other people get.	
	If an illness is ‘going around’, I will get it.	
	I am more prone to gout, hyperuricemia or other kidney diseases than people around me (reverse-scored)	
Environmental concerns	You often feel at one with the natural world around you.	Tan et al. (139)
	You recognize and appreciate the wisdom of other creatures	
	You can imagine yourself as part of a larger life cycle process.	
Familiarity	I know this nutritional product well and am familiar with this nutritional brand.	Gandhi and Ubba (145)
	I am very familiar with the nutritional products in this brand.	
Connection of nature	I am very aware of environmental issues.	Individuals’ connection (146)
	I always think about how my actions affect the environment.	
	I think a lot about the suffering of animals.	
Sustainable purchase intention	When you learnt about the above dietary supplements, would you consider purchasing this product on an ongoing basis?	Rausch and Kopplin (134)

## 4 Experiment 1: main effects of purine labeling of dietary supplements and consumers’ sustainable purchase intentions

### 4.1 Experimental design

We designed a one-way ANOVA experiment (purine labeling: yes vs. no) to exploring the effect of purine labeling of dietary supplements on consumers’ sustainable purchases intentions. We randomly summoned 200 subjects to participate in the experiment on a professional data collection platform, as shown in Table 4 and asked subjects whether they knew the meaning and risk hazards of purines. As 16 subjects were excluded due to strong consistency in questionnaire completion and an unclear understanding of the meaning of purine labeling, the effective sample size was 184. We randomly assigned all subjects to the dietary supplement sales scenario and divided them into a purine group (93) and a conventional group (91).

*Experimental procedure*: We guided all subjects to recall imagining that they had recently recovered from a serious illness, had weakened body resistance, and were selecting medicines in a large pharmacy where they would buy some dietary supplements. Immediately thereafter, we showed the dietary supplements with purine labeling to the study subjects in the purine group; the regular dietary supplements were shown to the study subjects in the conventional group. Immediately thereafter, we asked subjects about sustainable purchase intention measurement questions, such as, “When you learn about the dietary supplement mentioned above, would you consider purchasing the product?” (1 = strongly disagree, 7 = strongly agree) (134). Finally, we collected demographic information about the subjects.

### 4.2 Experimental results

*Main effects test*: We conducted a one-way ANOVA with the purine labeling of dietary supplements (purine group vs. conventional group) as the independent variable and consumers’ sustainable purchase intention as the dependent variable. The experimental results showed that the sustainable purchase intention of consumers in the purine group (M = 4.86, SD = 0.685) was significantly higher than that of the conventional group (M = 4.56, SD = 0.718), and *F*(1, 182) = 8.396, *p* = 0.004. It can be seen that consumers show higher purchase intention for dietary supplements containing purine labeling, which verifies hypothesis H1.

*Control variable analysis*: Given that Mostafa (135) study found that a consumer’s gender is an important influence on a consumer’s sustainable purchases intentions. Therefore, we used gender as a control variable and performed ANOVA. The experimental results showed that gender had no significant effect on consumers’ sustainable purchase intention [*F*(1,182) = 0.049, *p* = 0.825]. Therefore, gender has no significant effect on experiment results, testing hypothesis H1.

### 4.3 Discussion

We analyzed in Experiment 1 that the purine labeling of dietary supplements has a significant effect on consumers’ sustainable purchase intention to test Hypothesis H1. Specifically, consumers are more inclined to purchase dietary supplements with purine labeling than conventional dietary supplements. This may be due to the fact that modern consumers pay more attention to health management, and purine labeling enables these consumers to determine whether the product meets their health needs quickly, thus enhancing consumers’ purchase intention. In addition, we excluded the effect of gender on the experimental results in Experiment 1, which enhanced the accuracy of the study. Despite the above findings in Experiment 1, Experiment 1 did not further explore the internal mechanisms and boundary conditions between the purine labeling of dietary supplements and consumers’ sustainable purchase intentions. Therefore, we introduced health consciousness as a mediating variable in Experiment 2 to explore the mediating role of health consciousness.

## 5 Experiment 2: the mediating role of health consciousness

### 5.1 Experimental design

Experiment 2 aimed to investigate the mediating role of health consciousness on purine labeling of dietary supplements and consumers’ sustainable purchase intentions. We designed a one-way ANOVA experiment and randomly called 300 subjects to participate in the experiment by asking whether the subjects were aware of the meaning and risk hazards of purines in a professional data collection platform, as shown in Table 4. Since 25 subjects were excluded due to strong consistency in questionnaire completion and lack of knowledge about the meaning and hazards of purines, the effective sample size was 275. We randomly assigned all subjects to dietary supplement sales scenarios and divided them into a purine group (143) and a conventional group (132).

*Experimental procedure*: All subjects were instructed to imagine needing a dietary supplement to replenish their micronutrient depletion after a stressful work day. We presented a purine-labeled dietary supplement to the subjects in the purine group and a conventional dietary supplement to the subjects in the conventional group. Immediately following this, we asked subjects questions about measures of health consciousness, such as “Do you agree that you often reflect on your health? “(1 = strongly disagree, 7 = strongly agree) (136). Immediately after this the subjects were required to answer questions about the measurement of consumers’ sustainable purchase intentions (134).

Given Saba and Di Natale (137) research findings, consumption habits enhance consumers’ motivation to spend. Therefore, we need to control for consumption habits. We asked subjects, “Do you choose to buy regular dietary supplements out of habit? “(1 = strongly disagree, 7 = strongly agree) (137). Finally, we collected demographic information related to the subjects.

In view of the findings of De Canio et al. (138), environmental concerns play a mediating role in food labeling and consumers’ sustainable purchase intention. Therefore, to further enhance the accuracy of the experimental results of this study, we included environmental concerns as a surrogate explanatory variable for health consciousness. We collected participants’ measurement questions on environmental concerns (1 = strongly disagree, 7 = strongly agree) (139).

### 5.2 Experimental results

*Main effects test*: We conducted one-way ANOVA with the purine labeling of dietary supplements (purine group vs. conventional group) as the independent variable and consumer sustainable purchase intention as the dependent variable. The experimental results showed that consumers’ sustainable purchase intention (M _Purine group_ = 5.347, SD _Purine group_ = 1.541; M _Conventional group_ = 4.368, SD _Conventional group_ = 1.6), *F*(1, 273) = 26.67, *p* < 0.001. It can be seen that consumers showed higher purchase intention for dietary supplements containing purine labeling compared with conventional dietary supplements, which verified hypothesis H1.

**Figure 3:**
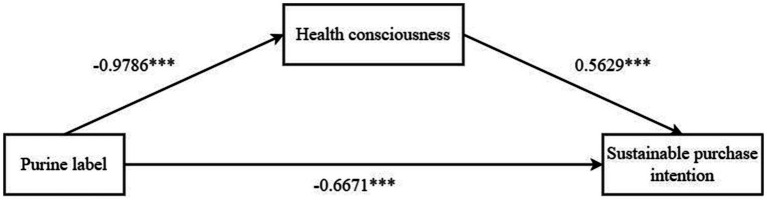
Path coefficient of the mediating effect of health consciousness. * * * *p* < 0.001, * * *p* < 0.01, * *p* < 0.05.

*Mediating effects test*: We used purine labeling of dietary supplements as the independent variable, consumers’ sustainable purchase intention as the dependent variable, and health consciousness as the mediating variable. We used Process Model 4 to test the mediating effect of health consciousness. The experimental results found that the purine labeling of dietary supplements-health consciousness-consumers’ sustainable purchase intention has a significant mediating effect (*β* = −0.5508, SE = 0.1284, 95% CI = [−0.8179~−0.3168]). Among them, purine labeling had a significant effect on consumers’ sustainable purchase intention (β = −0.6671, *p* < 0.001); health consciousness had a significant positive effect on consumers’ sustainable purchase intention (β = 0.5629, *p* < 0.001); and purine labeling had a positive effect on health consciousness (β = −0.9786, *p* < 0.001). It can be seen that health consciousness is a mediator of purine labeling and consumers’ sustainable purchase intention, testing hypothesis H2. As shown in Figure 3.

*Control variable analysis*: In view of Saba and Di Natale (137) research findings, consumption habit is an influential factor affecting consumers’ sustainable purchase intentions. Therefore, we conducted an analysis of covariance with consumption habit as covariates. The results showed that consumption habit had no significant effect on consumers’ sustainable purchase intention between purine labeling groups [*F*(1, 273) = 6.376, *p* = 0.002]. Therefore, the influence of consumption habits on the experimental results can be excluded, and hypothesis H1 can be tested again.

*Alternative explanations were excluded*: Referring to the method of Tan et al. (139), we took environmental concerns as the mediating variable and used Process Model 4 to analyze the mediating effect of environmental concerns. The results showed that environmental concerns had no significant mediating effect between purine labeling and consumers’ sustainable purchase intention (*β* = −0.1095, SE = 0.1018, 95%CI = [−0.3308~0.0651]). Therefore, there was no alternative explanation effect of environmental concern, which again verified hypothesis H2.

### 5.3 Discussion

We verified the mediating role of health consciousness on purine labeling and consumers’ sustainable purchase intentions in Experiment 2. Specifically, health consciousness enhances consumers’ attention to purine labeling and promotes their focus on sustainability choices. At the same time, we excluded the influence of consumption habits on the experimental results to enhance the robustness and scientific validity of the study. Despite the above findings in Experiment 2, Experiment 2 did not further explore whether there is a moderating effect between purine labeling and consumers’ sustainable purchase intention. Therefore, we introduced disease threat as an interaction variable in Experiment 3 to discuss the impact of the interaction effect of disease threat and purine labeling on consumers’ sustainable purchase intention.

## 6 Experiment 3: the interaction effect of disease threat and purine labeling

### 6.1 Experimental design

Experiment 3 aimed to verify the effect of the interaction of disease threat and purine labeling on consumers’ sustainable purchase intentions. We designed a 2 (purine labeling: purine group vs. conventional group) × 2 (disease threat: high vs. low) ANOVA experiment, and randomly called 500 subjects to participate in the experiment, a professional data collection platform. The demographic information is shown in Table 4. We randomly assigned all subjects to dietary supplement sales scenarios and divided them into a purine group (222) and a conventional group (278).

*Experimental procedure*: We asked all subjects to imagine that they were purchasing goods in a large pharmacy, and we then assigned the subjects in the purine group into two groups: high and low disease threat. We presented all subjects in the purine group with dietary supplements containing purine labeling. For the high-threat group, we told them: “Recently, there has been a new coronary epidemic in your area, and the number of infected people is increasing day by day, so the immunocompromised people need to take nutrient supplements.” For the low-threat group, we told them: “Recently, a new tertiary hospital has been constructed in the vicinity of your area, which has made it more convenient to go to the doctor in your daily life.” However, we showed the conventional dietary supplements for all subjects in the conventional group, in which the manipulation for disease threat was consistent with the purine group. Immediately after, we asked the subjects about measuring sustainable purchase intentions.

Given Kuang et al. (140) findings, emotions are an important influence on consumers’ sustainable purchase intentions. Therefore, using emotion as a control variable, we asked subjects, “When you see the above material information, do you agree that your emotions are positive (happy, joyful, blissful)?” (1 = strongly disagree, 7 = strongly agree) (140). Finally, we collected demographic information related to the subjects.

### 6.2 Experimental results

*Main effects test*: We conducted a one-way ANOVA with the purine labeling of dietary supplements (purine group vs. conventional group) as the independent variable and consumers’ sustainable purchase intention as the dependent variable. The experimental results show that the sustainable purchase intention of consumers in the purine group (M = 5.88, SD = 0.829) is significantly higher than that of the conventional group (M = 4.76, SD = 1.193), *F*(1, 498) = 140.149, *p* < 0.001. It can be seen that the consumers show higher purchase intention for dietary nutrients containing purine labeling, which verifies hypothesis H1.

**Figure 4:**
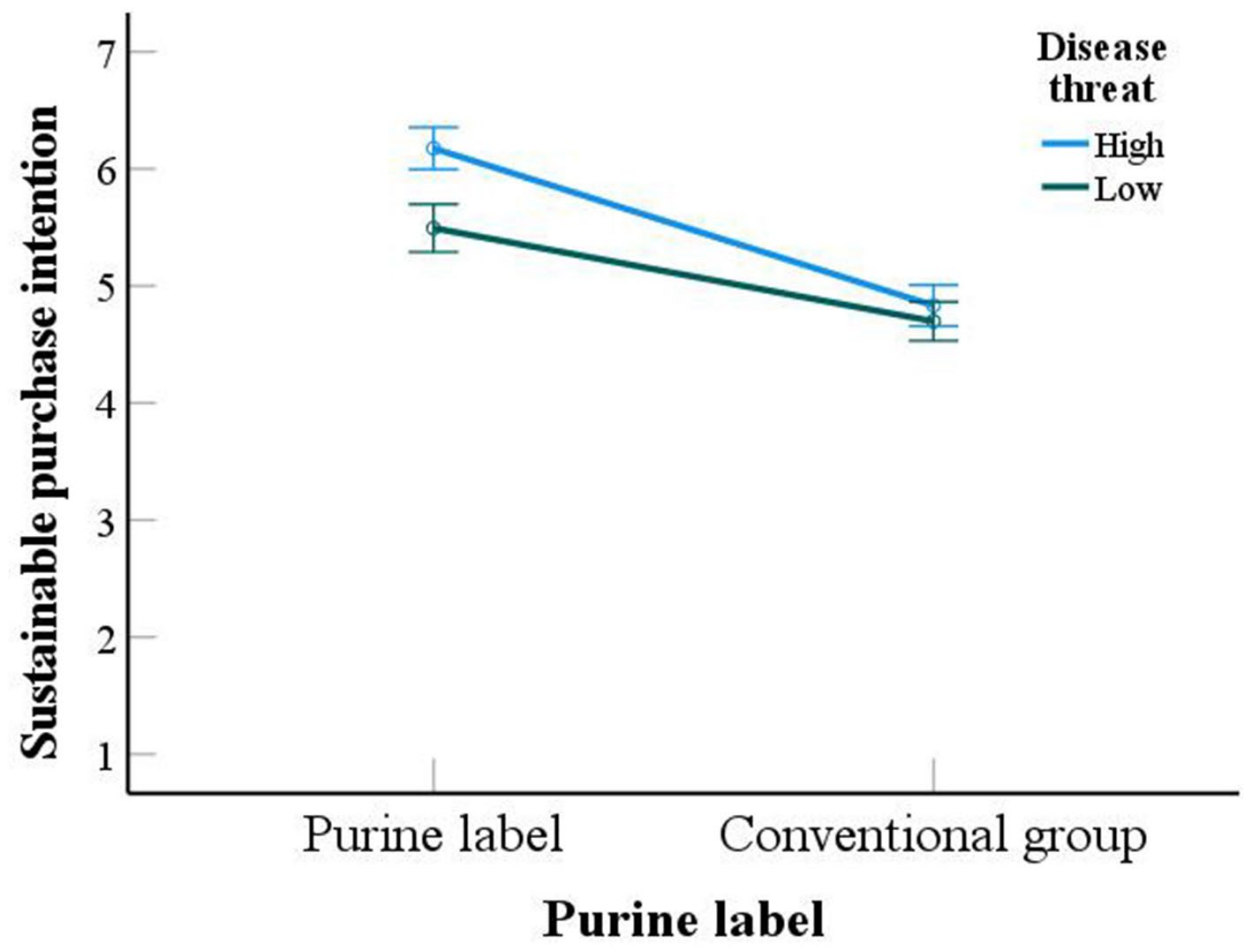
Interaction of disease threat and purine label.

*Interaction effect analysis*: We used purine labeling (purine group Vs. conventional group) as the independent variable, consumers’ sustainable purchase intention as the dependent variable and disease threat as the interaction variable. We used two-way ANOVA to test this. The results of the experiment showed that the interaction of disease threat and purine labeling had a significant effect on consumers’ sustainable purchase intention [*F*(1,498) = 8.71, *p* = 0.003]. Disease threat has a significant effect on consumers’ sustainable purchase intention [*F*(1,498) = 19.416, *p* < 0.001]. Purine labeling has a significant effect on consumers’ sustainable purchase intention [*F*(1,498) = 133.447, *p* < 0.001]. Specific details are shown in Figure 4 to test hypothesis H3.

Based on the findings of Liang et al. (26), we used emotion as a covariate for the control variable test. Based on the results of the analysis of covariance showed that emotion had no significant effect on the experimental results [*F*(1,497) = 66.765, *p* < 0.001]. Therefore, the control variable emotion can be excluded, and hypothesis H3 can be tested again.

### 6.3 Discussion

We verified the effect of the interaction of disease threat and purine labeling on consumers’ sustainable purchase intentions in Experiment 3. Specifically, dietary supplements containing purine labeling were more popular among consumers compared to dietary supplements in the conventional group. At the same time, we elaborated that emotion had no significant effect on the experimental results. Despite the above findings in Experiment 3, Experiment 3 had not been analyzed from the perspective of consumers’ disease fear. Therefore, we introduced the fear of risk as a moderating variable in Experiment 4 to analyze the moderating effect of fear of risk on the purine labeling of dietary supplements and consumers’ sustainable purchase intentions.

## 7 Experiment 4: the moderating effect of fear of risk

### 7.1 Experimental design

Experiment 4 aimed to verify the moderating effect of fear of risk on the purine labeling of dietary supplements and consumers’ sustainable purchase intentions. We designed a 2 (purine labeling: purine group vs. conventional group) × 2 (fear of risk: high vs. low) variance experiment and randomly called 300 subjects to participate in the experiment by asking them if they knew the meaning of purine and the risk hazards in a professional data collection platform. Since 30 subjects were excluded because they did not know the meaning of purine, the effective sample size was 270. The demographic information is shown in Table 4. We randomly assigned all subjects to dietary supplement sales scenarios and divided them into a purine group (132) and a conventional group (138).

*Experimental procedure*: All subjects were instructed to imagine that they had recently needed to purchase a nutritional supplement for their personal health care needs, and they traveled to a large pharmacy to make their selections. During the selection process, the researcher showed the study subjects in the purine group a dietary supplement with a purine labeling; the study subjects in the conventional group were shown a conventional dietary supplement. Immediately after, subjects were asked to answer questions measuring fear of risk, e.g., I become nervous or anxious when I see disease-related information on dietary supplements (1 = Strongly Disagree, 7 = Strongly Agree) (141); then, subjects were asked to answer questions measuring sustainable purchase intentions. Finally, we collected demographic information related to the subjects.

### 7.2 Experimental results

*Main effects test*: We used the purine labeling of dietary supplements (purine group vs. conventional group) as the independent variable and consumers’ sustainable purchase intention as the dependent variable and conducted a one-way ANOVA. The results showed that consumers’ sustainable purchase intentions (M _purine group_ = 6.67, SD _purine group_ = 0.473; M _conventional group_ = 6.1, SD _conventional group_ = 1.234), *F*(1, 268) = 24.279, *p* < 0.001. It can be seen that, compared with conventional dietary supplements, consumers showed a higher willingness to purchase, testing hypothesis H1.

**Figure 5:**
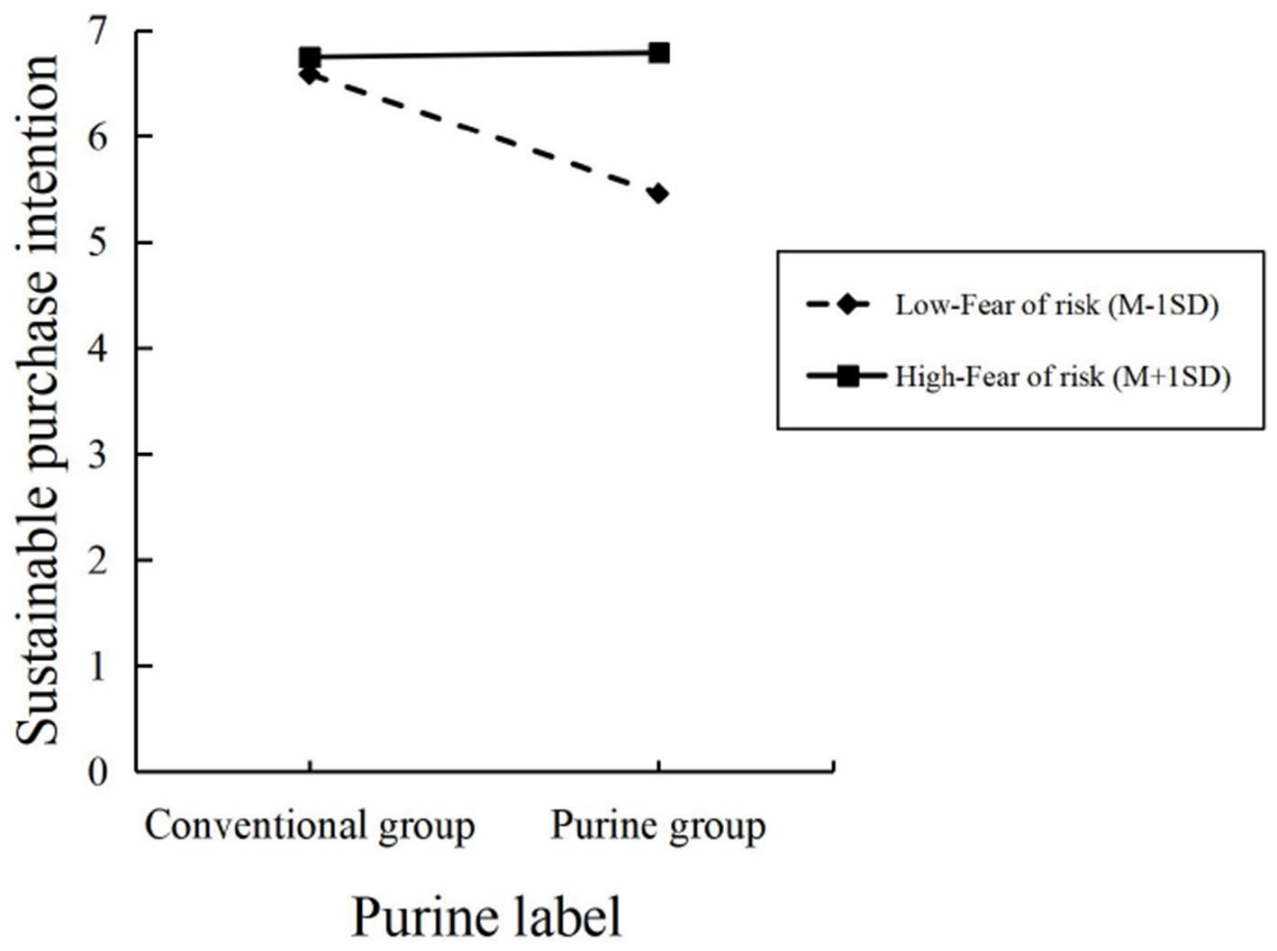
Interaction of fear of risk and purine labeling.

*Moderating effects test*: We used the purine labeling of dietary supplements (purine group Vs. conventional group) as the independent variable, consumers’ sustainable purchase intention as the dependent variable, and fear of risk as the moderating variable. We used Process Model 1 for the moderating effect of fear of risk. The experimental results show that the interaction of fear of risk and purine labeling has a significant effect on consumers’ sustainable purchase intentions (*β* = 0.5812, SE = 0.099, *p* < 0.001); fear of risk has a significant effect on consumers’ sustainable purchase intentions (β = 0.3783, SE = 0.0497, *p* < 0.001); purine labeling has a significant effect on consumer sustainable purchase intention (β = −0.5406, SE = 0.099, *p* < 0.001). It can be seen that fear of risk has a significant moderating effect on the purine labeling of dietary supplements and consumers’ sustainable purchase intentions, testing hypothesis H4, as shown in Figure 5.

### 7.3 Discussion

We verified in Experiment 4 that fear of risk has a significant moderating effect on the purine labeling of dietary supplements and consumers’ sustainable purchase intentions. Specifically, sustainable purchase intentions were consistently higher for consumers with high fear of risk, regardless of purine labeling, compared to the group with low fear of risk. In the conventional group, the difference in sustainable purchase intentions between low-risk-phobic and high-risk-phobic consumers was slight. However, when purine labeling is introduced, a more significant gap emerges. Specifically, low-risk-phobic consumers exhibited a significant decrease in sustainable purchase intentions, while high-risk-phobic consumers maintained their purchase intentions. This suggests that purine labeling may deter lower fear of risk consumers more than higher fear of risk consumers. Consumers with higher risk sensitivity appear less negatively affected by purine labeling, possibly because they are more concerned about health or consumption-related risks.

## 8 Experiment 5: mediating effects of perceived gout susceptibility

### 8.1 Experimental design

Experiment 5 was designed to test the mediating effect of perceived gout susceptibility on the purine labeling of dietary supplements and consumers’ sustainable purchase intention. We designed a single-factor inter-subject variance experiment (purine labeling: purine group vs. conventional group) and asked whether they knew the meaning and risk of purine through a professional data collection platform. Three hundred subjects were randomly recruited to participate in the experiment. 19 subjects were excluded because the meaning of purines was unknown, resulting in an effective sample size of 281. Demographic information is presented in Table 4. We randomly assigned all subjects to the dietary supplement sales scenario and divided them into purine (140) and conventional groups (141).

*Experimental procedure*: All participants were instructed to imagine that they had recently received a lecture on gout and hyperuricemia and, in the process, learned about the importance of choosing appropriate dietary supplements. Therefore, participants were prepared to go to the pharmacy to select the appropriate supplement that was pressed. During selection, participants in the purine group were shown a purine-labeled dietary supplement; Participants in the usual care group received conventional dietary supplements. Immediately following, participants were asked to answer the perceived gout susceptibility scale, which was adapted from the perceived illness susceptibility scale developed by Duncan et al. (142) (1 = strongly disagree, 7 = strongly agree). For example, my immune system protects me from most illnesses that other people get. Then, the participants were asked to answer a question measuring the sustainable purchase intention. Cai et al. (143) and Wu et al. (144) found that familiarity often mediated the effect of product labels on consumers’ sustainable purchase intention. Based on this, we included familiarity as an alternative explanatory variable on a scale derived from Gandhi and Ubba (145) (1 = strongly disagree, 7 = strongly agree). Finally, we collected the demographic information of the subjects.

### 8.2 Experimental results

*Main effect test*: We conducted a one-way ANOVA with the purine labeling of the dietary supplement as the independent variable and the consumer’s sustainable purchase intentions as the dependent variable. The results showed that the sustainable purchasing intention of the purine group (M = 5.49, SD = 1.462) was significantly higher than that of the conventional group (M = 4.38, SD = 1.019), *F*(1, 279) = 53.887, *p* < 0.001. Thus, it can be seen that consumers show higher purchase intention for dietary nutrients containing purine labeling, verifying hypothesis H1.

**Figure 6:**
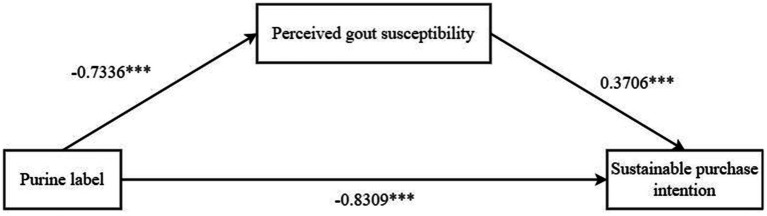
Mediating effect path coefficient of perceived gout susceptibility. * * * *p* < 0.001, * * *p* < 0.01, * *p* < 0.05.

*Mediation effect test*: We used Process Model 4 to test the mediating role of perceived gout susceptibility using the purine labeling of dietary supplements as the independent variable, perceived gout susceptibility as the mediating variable, and consumers’ sustainable purchase intention as the dependent variable. The results showed that the purine labeling of dietary supplements had a significant impact on consumers’ sustainable purchase intention (*β* = −0.8309, *p* < 0.001). The purine labeling of dietary supplements had a significant effect on the perceived susceptibility to gout (β = −0.7336, *p* < 0.001). Perceived gout susceptibility had a significant impact on consumers’ sustainable purchase intention (β = 0.3706, *p* < 0.001). Overall, perceived gout susceptibility had a mediating effect on purine labeling and consumers’ sustainable purchase intention (β = −0.2719, SE = 0.079, 95% CI = [−0.44~−0.1292]), as shown in Figure 6 to test hypothesis H5.

*Alternative explanations were excluded*: We examined the mediating effect of familiarity by substituting familiarity for perceived gout susceptibility as a mediating variable. Referring to the study of Tan et al. (139), we used Process Model 4 to examine the mediating effect of familiarity. The results showed that familiarity had no significant mediating effect on purine labeling and consumers’ sustainable purchase intention (β = −0.2169, SE = 0.1743, 95% CI = [−0.5599~0.1335]). Therefore, we can rule out the alternative explanatory role of familiarity, again validating hypothesis H5 and enhancing the accuracy of the experiment.

### 8.3 Discussion

We demonstrated in Experiment 5 that perceived gout susceptibility has a mediating effect on purine labeling and consumers’ sustainable purchase intention, testing hypothesis H5. At the same time, we excluded the alternative explanatory role of familiarity and enhanced the robustness of the experiment. Specifically, this mediating effect comes from consumers’ pursuit of health and self-protection consciousness. Through purine labeling, consumers can better manage their diet and reduce gout risk, enhancing their sustainable purchase intention. Despite the above findings of Experiment 5, Experiment 5 did not explore the internal motivation of consumers’ sustainable purchase from the perspective of consumption sustainable consumption.

The raw materials of dietary nutritional supplements are mainly from natural species, such as protein powder. If soybeans are used as raw material because soybeans themselves contain relatively high purine contain, the purine content of this kind of protein powder will also be high. Some nutritional supplements are made from animal offal, seafood, etc. In the production process, dietary nutritional supplements will also contain purines if additives and excipients containing purines are used or factors such as production environment and process lead to the introduction of purines. Therefore, purine labels can effectively reflect the content of purine in products and stimulate consumers’ sustainable purchase intention. Therefore, dietary nutritional supplements with low purines may bring lower environmental damage and lower damage to animal viscera, seafood, high-protein plants, etc. At the same time, low-purine or zero-purine dietary supplements can reduce the intake of purine, reduce the incidence of gout or hyperuricemia, reduce the consumption of medical resources, and improve the quality of life of the population, which is in line with the goal of sustainable consumption to promote human long-term well-being.

Therefore, we introduced the connection of nature as an independent variable in Experiment 6 to analyze its moderating effect of connection of nature on purine label and consumer sustainable purchase intention.

## 9 Experiment 6: the moderating effect of connection of nature

### 9.1 Experimental design

This study aimed to verify the interaction of natural associations and purine labeling on consumers’ sustainable purchase intention. We designed a 2 (purine labeling: purine group vs. conventional group) × 2 (connection of nature: high vs. low) variance experiment in Experiment 6. We immediately recruited 300 participants on a professional data collection platform and asked them whether they knew the meaning and risks of purine. Since 24 subjects were removed due to failure of the attention test and ignorance of purine meaning, the effective sample size was 276. Demographic information is presented in Table 4. We randomly assigned all subjects to the dietary supplement sales scenario and divided them into the purine group (145) and the conventional group (131).

*Experimental procedure*: All participants were instructed to imagine that they had recently been in relatively poor physical condition due to the impact of the COVID-19 pandemic and urgently needed a dietary supplement. During selection, participants in the purine group were shown a purine-labeled dietary supplement; Participants in the conventional group received conventional dietary supplements. Next, participants were asked to answer the connection of nature measurement question, which was adapted from the Individuals’ Connection (146) research scale (1 = strongly disagree, 7 = strongly agree), e.g.: When you see the dietary supplements mentioned above, I think a lot about animal suffering. Then, the participants were asked to answer a question measuring the sustainable purchase intention. Finally, we collected the demographic information of the subjects.

### 9.2 Experimental results

*Main effect test*: We conducted a one-way ANOVA with the purine labeling of the dietary supplement as the independent variable and the consumer’s sustainable purchase intention as the dependent variable. The results showed that the consumer’s sustainable purchase intention (M _purine group_ = 5.29, SD _purine group_ = 1.699; M _conventional group_ = 4.49, SD _conventional group_ = 1.643), *F*(1,274) = 15.787, *p* < 0.001. Thus, it can be seen that consumers show higher purchase intention for dietary supplements with purine labels, which validates hypothesis H1.

**Figure 7:**
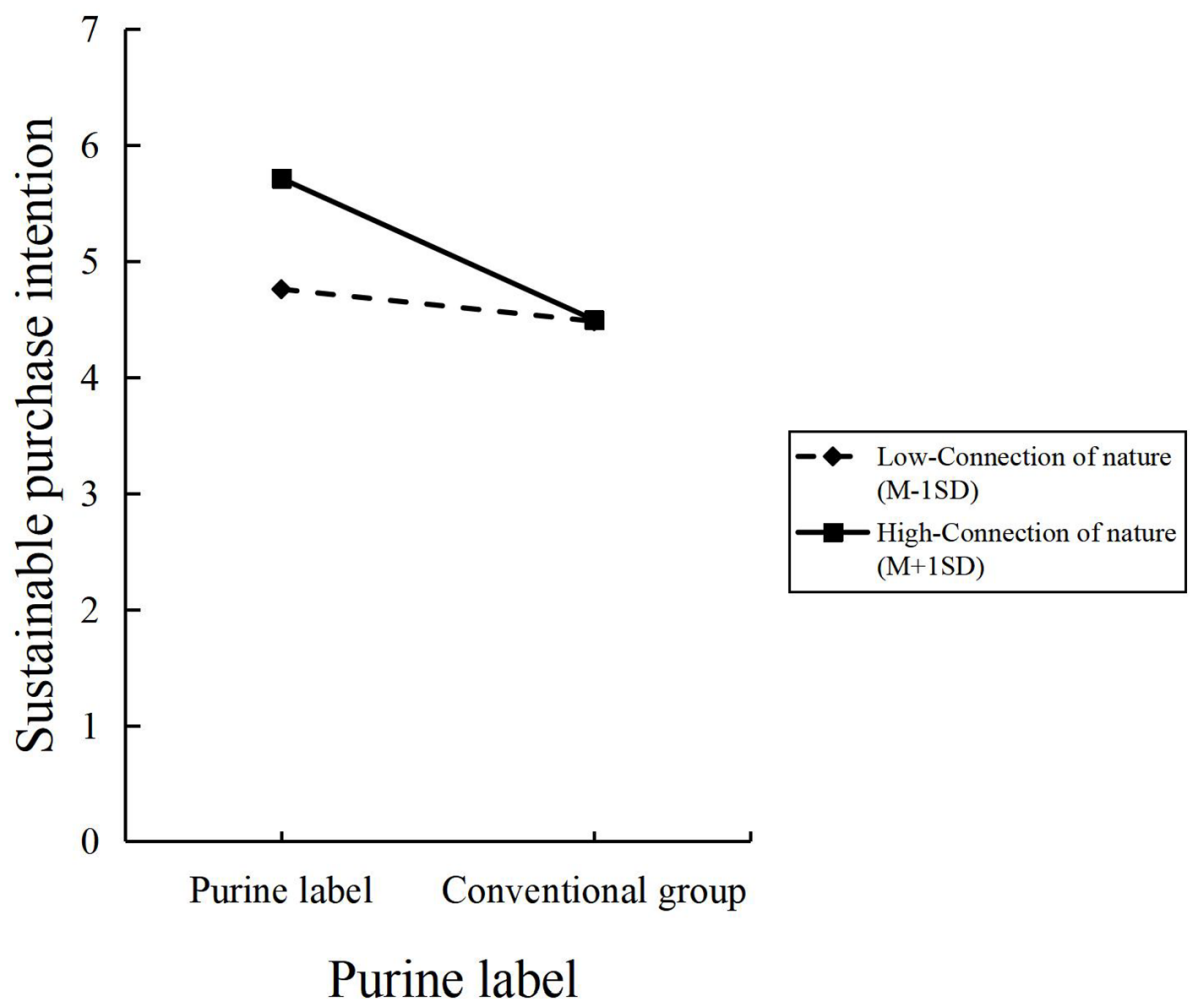
Interaction effects of connection of nature and purine labels.

*Moderation effect test*: We used Process Model 1 to test the moderating effect of the connection of nature using the connection of nature as moderator variables, purine labeling of dietary supplements as independent variables and consumers’ sustainable purchase intention as dependent variables. The results showed that purine labeling of dietary supplements had a significant impact on consumers’ sustainable purchase intention (*β* = −0.7485, *p* < 0.001). The connection of nature had a significant impact on consumers’ sustainable purchase intention (β = 0.2148, *p* = 0.0118). The interaction between purine labeling and connection of nature had a significant impact on consumers’ sustainable purchase intention (β = −0.3996, *p* = 0.0188, 95% CI = [−0.7325~−0.0667]). Therefore, The connection of nature have a significant impact on the purine labeling of dietary supplements and consumers’ sustainable purchase intention, as shown in Figure 7 to test hypothesis H6.

### 9.3 Discussion

In Experiment 6, we demonstrated the significant effects of connection of nature on purine labeling and consumers’ sustainable purchase intention of dietary supplements. Specifically, in the purine labeling condition, consumers often show higher sustainable purchase intentions when they have higher connection of nature. In the high connection of nature condition, consumers are more likely to buy the dietary supplements with purine labeling.

## 10 Discussion

### 10.1 Theoretical implications

This study has several important theoretical implications. First, this study found that purine labeling of dietary supplements had a positive effect on consumers’ sustainable purchase intentions. In previous studies, nutritional labeling has been identified as an effective factor influencing consumers’ consumption decisions (147–149). However, few studies have linked purine labeling to consumer behavior. For example, sugar-free labeling can effectively enhance consumers’ consumption motivation (25). Although the above studies have focused on the relationship between nutrition labeling and consumer behavior, the impact of purine labeling of dietary supplements on consumer’s sustainable purchase intention has not been deeply studied. Therefore, the present study responds to the call for recent studies highlighting the important impact of nutritional indicators of dietary supplements (150, 151). By integrating these factors into the effect of purine labeling of dietary supplements on consumers’ sustainable purchase intention, this study provides new perspectives for research in consumer nutrition consumption. On the one hand, this study expands the literature related to the purine labeling of dietary supplements and the sustainable consumption behavior of consumers; on the other hand, by highlighting new antecedents of consumers’ sustainable purchase intentions of dietary supplements, the findings provide *a priori* knowledge and a better explanation of how the purine labeling of dietary supplements may influence consumers’ sustainable purchase intentions.

Based on the Health Belief Model, this study aims to analyze the mediating role of health consciousness in how purported labeling of dietary supplements influences consumers’ sustainable purchase intentions. It has been shown that nutritional labeling plays an important role in consumers’ choice of nutritional supplements, especially in consumer consumption decisions and health consciousness (152, 153). However, the specific mechanisms by which purine labeling influences consumers’ sustainable purchase intentions have not been fully explored. Therefore, the present study further extends the relevant field of research by exploring the mediating role of health consciousness in dietary supplements and consumers’ sustainable purchase intentions. Although previous studies have supported the ability of health consciousness to influence consumers’ choice of dietary supplements (154, 155), the specific mediating role of health consciousness between purine labeling and sustainable purchase intentions is not known. Therefore, this study is the first attempt to validate the mediating role of health consciousness in this relationship, revealing that purine labeling influences consumers’ sustainable purchase intentions for dietary supplements by enhancing their health consciousness and, consequently, their willingness to purchase dietary supplements. This finding has important theoretical implications for the development of more targeted product labeling and marketing strategies.

This study further identifies the moderating role of disease threat and fear of risk in the impact of purine labeling on consumers’ sustainable purchase intentions. It has been shown that individuals’ perceived health threat and fear of potential disease risk significantly affect their sensitivity to relevant food labelings (156, 157). On the one hand, in the face of health problems caused by high purine content, such as gout and cardiovascular disease, consumers with higher health consciousness are more likely to be concerned about the purine labeling of dietary supplements. This concern prompts them to more prudently choose products that meet their health needs, thus enhancing their sustainable purchase intention. On the other hand, consumers with a lower perceived disease threat or a lower fear of risk may not prioritize the impact of purine labeling, and thus their moderating effect on sustainable purchase intentions may be weaker.

In addition, fear of risk reinforces consumers’ health behaviors decision-making process (158). When consumers perceive a high risk of disease threat, they tend to take preventive measures (159), such as choosing healthier dietary supplements. Purine labeling become important reference information at this point, and consumers will make more rational purchase decisions based on the labels to avoid future health risks. Thus, fear of risk and disease threat together play a key moderating role in the way purine labeling affects consumers’ sustainable purchase intention. This finding expands research in the field of dietary supplements and provides new perspectives for understanding consumers’ purchasing behavior in the face of health risks.

### 10.2 A dual drive model for health and the environment

As an important health information signal, the purine label of dietary supplements has a significant impact on consumers’ health perception and purchase behavior. Purines are a potential health risk factor, especially for consumers with gout or hyperuricemia, which may be aggravated by excessive intake. Therefore, the existence of purine labeling is not only a reflection of information transparency, but also a direct protection of consumer health. Consumers’ perceived gout susceptibility as the key mediating variable may have reinforced the effect of purine labeling on sustainable purchase intention. Specifically, when consumers perceive that they are at higher risk of gout, they may be more inclined to choose low-purine or no-purine dietary supplements, significantly increasing the sustainable purchase intention. In addition, health consciousness, as another mediating variable, also plays an important role in this relationship. Consumers with higher health consciousness are more likely to pay attention to purine labeling, thereby forming health preferences and sustainable purchasing behaviors. In general, purine labeling significantly enhanced consumers’ sustainable purchase intention by arouse their health risk cognition and health consciousness, which provided theoretical support for health-driven consumption decisions.

Environmental drivers also play an important role in the formation of consumers’ sustainable purchase intention. As a psychological state, the connection of nature reflects consumers’ sense of dependence on the natural environment and environmental responsibility. Studies have shown that consumers with high connection of nature are more likely to choose products with sustainability and environmental features. In the context of purine labeling of dietary supplements, the connection of nature can further influence consumers’ purchase intention by strengthening their environmental attitude and sustainable consumption behavior. For example, consumers with a high connection of nature may be more concerned about a product’s environmental footprint and tend to choose products that are sustainably produced and clearly labeled with natural ingredients in their packaging. In addition, disease threat and fear of risk, as moderating variables, may also further moderate the effect of purine labeling on sustainable purchase intention by reinforcing consumer perceptions of health and environmental risks. For example, when consumers perceive the threat of gout, they may not only choose low-purine products but also be more inclined to support environmental policies and sustainable businesses. Therefore, environmental drivers significantly influence consumers’ sustainable purchase intentions by modulating their environmental attitudes and risk perceptions.

The dual driving effect of health and environment on consumers’ sustainable purchase intention has important theoretical significance. Firstly, from the theoretical level, this study extends the theoretical framework of sustainable purchase intention to include health and environmental factors in the analysis, and reveals the multi-dimensional mechanism by which purine labels affect consumer behavior. Traditional studies often focus on a single driver, such as health or the environment, but ignore the possible synergistic effect of both. This study revealed the complex interaction between health and environmental factors in sustainable purchase intention by introducing mediating variables such as perceived gout susceptibility and health consciousness, and moderating variables such as connection of nature and disease threat. Secondly, from a practical level, this study provides important guidance for food companies and policymakers. For example, companies can meet consumer needs driven by both health and the environment by designing clearer purine labels and strengthening the environmental characteristics of products. At the same time, policymakers should strengthen the supervision of food labels to ensure the accuracy and comparability of purine labeling, thereby protecting consumer rights and promoting sustainable consumption.

### 10.3 Practical implications

This study has several practical implications. First, this study demonstrates that under the influence of purine labels, consumers will pay more attention to the health attributes of products when making purchase decisions, especially for consumers with higher health consciousness, who will analyze the health risk information conveyed by purine labeling more deeply. Recent studies have found that more health consciousness, consumers are more willing to buy organic green food (160). This suggests that companies should focus on the precise design of purine labeling in product marketing, clearly highlighting the health attributes of the product, especially for consumer groups with higher health consciousness. Through effective health messaging, consumers can be helped to better understand product ingredients and their possible health effects (161), thereby increasing their sustainable purchase intentions, which is crucial to the long-term market competitiveness of the product.

Second, this study also reveals the moderating role of disease threat in the relationship between purine labeling and consumer purchase intention. The experimental results show that when consumers perceive a higher disease threat, they are more inclined to pay attention to the health risk cues of purine labeling, which further increases the sustainable purchase intention of the product. For consumer groups with higher health risks, especially those with diseases related to purine metabolism, such as gout, companies should pay more attention to the communication of labeling information in the marketing process of dietary supplements to strengthen consumers’ perception of health risks. This labeling strategy based on risk perception can help enhance consumer consciousness and trust in the product and promote market expansion, especially in the special population market.

Finally, the study found a significant effect of fear of risk on consumers’ sensitivity to purine labeling, which further highlights the psychological response of consumers when faced with specific health risks (162, 163). By combining strong risk education with labeling information, companies can take advantage of consumers’ fear of risk and raise their awareness of product ingredients and health risks. This not only promotes consumer recognition and trust in dietary supplements but also establishes the company’s healthy brand image in the market, enhances the product’s sustainable purchase intention (21). In product promotion, through accurate market segmentation, differentiated labeling strategies for high-risk groups can not only effectively convey health risk information but also enhance the market influence and long-term competitiveness of the product.

### 10.4 Policy implications

From the perspective of policymakers, this study explored the impact of purine labeling on consumers’ sustainable purchase intention and its policy implications, which provided a scientific basis for regulators to optimize labeling regulations, improve public health and promote the sustainable development of the food industry. It was found that purine labeling significantly affected consumers’ sustainable purchase intention by triggering their health consciousness and perceived gout susceptibility, and this effect showed significant differences under different levels of the connection of nature, disease threat, and fear of risk. These findings suggest that purine labeling is not only an important tool for consumer health management but also a key means for policymakers to achieve public health goals. The core of this study is to optimize the design and promotion of purine labeling through policy intervention to achieve win-win results among consumers, enterprises and society.

Regulatory agencies, such as the FDA and the European Food Safety Authority, establish clear regulations related to purine labeling, especially mandatory labeling measures for high-risk populations. First, regulators should promote the standardization and transparency of purine labeling in the food industry, requiring manufacturers to clearly purine labeling content and its potential health risks on product packaging, especially for high-risk groups such as gout patients and people with abnormal uric acid metabolism. Second, for specific high-risk groups, regulatory agencies can introduce high-purine warning labeling or low-purine recommendation labeling to help these groups quickly identify suitable products for them. Finally, regulatory agencies should establish a dynamic update mechanism to regularly revise the standards and requirements for purine labeling according to scientific research progress and public health needs. These policy measures will help consumers make more informed purchasing decisions but also help move the food industry in a healthier and more sustainable direction.

In addition, this study provides important implications for the role of policymakers in public health education and nutrition interventions. Policymakers can use a variety of channels, including social media, public health activities and school education, to popularize the meaning and interpretation of the purine labeling and improve the public’s attention and understanding of the purine labeling. For example, regulators could work with medical institutions and patient organizations to develop specialized health education programs for gout patients and those with high uric acid to help them better use purine labeling information. In addition, policymakers can also encourage companies to develop and promote low-purine products through tax incentives or subsidies, thereby reducing the health threat of high-purine foods to high-risk populations. These policy measures will contribute to a healthier and more sustainable environment for food consumption.

### 10.5 Research limitations and future research directions

Despite the above findings of this study, there are still some limitations. Firstly, the main limitations of this study are sample selection and external validity. The sample was mainly from China, which may limit the generalizability of the findings. Consumer patterns of dietary supplement use and interpretation of health labeling may differ significantly across cultures. Therefore, the results of this study may be more reflective of the behavior of Chinese consumers, and the results may be different in other cultural contexts, such as Western countries. Future studies could be cross-validated in Western countries to confirm the generalizability of the findings in this study. The sample in this study had some limitations in terms of age and educational level. Older adults were underrepresented in the sample, which may have limited our understanding of differences in behavior across age groups under the influence of purine labels. Considering that consumption behavior may be significantly influenced by health concerns, lifestyle habits, and other factors at different ages, future research should focus more on the diversity of samples, including a broader age range and educational background.

Second, the experimental design of this study is an operationalized way of defining and measuring health threats and risk fears. Although this study induced consumers’ health anxiety through specific situations in the experiment, these situations may not fully reflect the complexity of real-life situations. Consumers’ perceived disease threat and risk fear are the result of a multifactorial combination of factors. Future research should consider incorporating more contextual variables, such as social pressure, the influence of the consumption environment, and a longitudinal study design over a long period in order to capture the long-term effects of purine labeling on consumers’ behavior more comprehensively.

Third, the limitations of the data analysis methodology are of concern. This study relied heavily on laboratory-controlled settings and questionnaire data, which may have led to social desirability bias and self-reported limitations. Consumers may show inconsistent purchase intentions with their actual behavior in experimental situations. Future studies could incorporate behavioral data, such as actual purchase records, or use objective indicators, such as eye-tracking or field experiments (e.g., tracking real pharmacy purchase). This approach would validate the findings and enhance the reliability and authenticity of the results. Additionally, the study should explore the applicability of purine labeling in different product categories and market contexts further to extend the application and value of the study.

Fourth, this study only used “no purine” labeling, not other ones, to measure consumers’ sustainable purchase intention. This may lead consumers to choose the product for health reasons, resulting in limitations in the accuracy of the experiment. This is mainly because the experimental purpose of this study was to analyze whether the sustainable purchase intention of consumers would be affected by purine labeling. Therefore, future research can use the remaining labeling to analyze further the relationship between purine label types and consumer purchase intention.

Finally, the cross-sectional nature of the experiments presents another limitation. While the study provides valuable insights into consumers’ responses to purine labeling, it does not examine whether these effects are sustained over time. To address this, future research should employ longitudinal study designs to test habit formation and determine whether the behavioral changes induced by purine labeling are maintained in the long term. This would provide deeper insights into the potential of purine labeling as a tool for promoting public health and informed decision-making.

## 11 Conclusion

In this study, we systematically conducted six empirical experiments to comprehensively investigate consumers’ cognitive perception of purine labels and the mechanisms by which they influence sustainable purchasing behavior. Specifically, we first demonstrated that consumers’ willingness to purchase dietary supplements with purine labels is higher than that for conventional dietary supplements. Additionally, we found that health-conscious consumers are more likely to pay attention to the ingredients of dietary supplements and their associated health risks, which enhances their understanding of purine labeling and promotes their sustainable purchase intention. Furthermore, we explained how disease threat amplifies the impact of purine labels on consumers’ purchasing intentions by increasing their attention to the health risks of products. Disease threat increases consumers’ concerns about health risks, thereby intensifying the influence of purine labeling on their purchasing intentions. Consumers under high disease threats are likelier to exhibit strong and sustainable purchase intentions to prevent or mitigate disease risks. Fourth, we discovered that when consumers face health risks related to purine metabolism, such as gout, fear of risk may significantly increase their sensitivity to product label information and deepen their attention to dietary supplement ingredients, thereby affecting their attention to purine labeling and sustainable purchasing intentions. Fifth, we found that when consumers perceive disease susceptibility, they trigger risk-avoidance behaviors, prompting them to seek information to reduce health risks actively. Purine labeling serve as external health information cues, and individuals with high susceptibility exhibit heightened sensitivity to purine labeling information, leading to increased purchasing intentions. Sixth, we found that the connection of nature amplifies the environmental signaling effect of purine labeling. Purine labeling content on dietary supplement packaging may imply low-pollution production processes, and consumers with high connection of nature are more willing to engage in purchase behavior.

The results of the six experiments collectively demonstrate that purine labels play a significant positive role in driving consumers’ sustainable purchasing behavior, and this influence is the result of the joint action of multiple health-related psychological mechanisms. Specifically, the experiments revealed how factors such as health consciousness, disease threat, perceived gout susceptibility, fear of risk, and connection of nature reinforce the effects of purine labeling through different pathways. Therefore, purine labeling, as a tool for conveying health information, have demonstrated significant practical application value in promoting consumers’ sustainable purchase behavior. Future research can further explore the effects of other health information labeling and extend these findings to application scenarios in different cultural and environmental contexts to provide more universally applicable theoretical support and practical recommendations for health marketing. Researchers and practitioners should also focus on optimizing label design and precise marketing strategies to release the potential value of purine labeling further, creating dual benefits for consumer health management and corporate sustainable development.

## Data Availability

The raw data supporting the conclusions of this article will be made available by the authors, without undue reservation.

## Footnotes

^1^ https://e.tb.cn/h.6jgxa18xvGdnVvF?tk=letEVXZFokr
